# Parent Cardiac Response in the Context of Their Child’s Completion of the Cold Pressor Task: A Pilot Study

**DOI:** 10.3390/children4110100

**Published:** 2017-11-21

**Authors:** Kaytlin Constantin, Rachel L. Moline, C. Meghan McMurtry, Heidi N. Bailey

**Affiliations:** 1 Department of Psychology, University of Guelph, Guelph, ON N1G 2W1, Canada; rmoline@uoguelph.ca (R.L.M.); cmcmurtr@uoguelph.ca (C.M.M.); hnbailey@uoguelph.ca (H.N.B.); 2 Pediatric Chronic Pain Program, McMaster Children’s Hospital, Hamilton, ON L8N 3Z5, Canada; 3 Children’s Health Research Institute, London, ON N6C 2V5, Canada; 4 Department of Paediatrics, Schulich School of Medicine & Dentistry, Western University, London, ON N6A 3K7, Canada

**Keywords:** pediatric pain, physiological responding, heart rate, heart rate variability, parent–child interactions

## Abstract

Parents’ ability to regulate their emotions is essential to providing supportive caregiving behaviours when their child is in pain. Extant research focuses on parent self-reported experience or observable behavioural responses. Physiological responding, such as heart rate (HR) and heart rate variability (HRV), is critical to the experience and regulation of emotions and provides a complementary perspective on parent experience; yet, it is scarcely assessed. This pilot study examined parent (*n* = 25) cardiac response (HR, HRV) at rest (neutral film clip), immediately before the cold pressor task (pre-CPT), and following the CPT (post-CPT). Further, variables that may influence changes in HR and HRV in the context of pediatric pain were investigated, including (1) initial HRV, and (2) parent perception of their child’s typical response to needle procedures. Time-domain (root mean square of successive differences; RMSSD) and frequency-domain (high-frequency heart rate variability; HF-HRV) parameters of HRV were computed. HR and HF-HRV varied as a function of time block. Typical negative responses to needle pain related to higher parental HR and lower HRV at rest. Parents with higher HRV at baseline experienced the greatest decreases in HRV after the CPT. Consequently, considering previous experience with pain and resting HRV levels are relevant to understanding parent physiological responses before and after child pain.

## 1. Introduction

Pain is defined as a subjective “unpleasant sensory and emotional experience associated with actual or potential tissue damage, or described in terms of such damage” [1] (p. 210). Children frequently experience pain as a result of routine medical care, including immunizations and venipunctures [2]. These are commonly feared medical procedures, with 63% of young children and adolescents reporting a fear of needles, and 7% of parents and 8% of children reporting needle fear as a main reason for immunization non-compliance [3]. Unmanaged pain and fear associated with needles can result in other potential consequences, such as greater pain in subsequent procedures, higher risk of experiencing fainting and seizures, more fearful memories of pain, and avoidance of preventative health care [4]. It follows that understanding the factors that affect children’s pain experience is necessary to reduce and prevent these short- and long-term consequences. 

### 1.1. The Role of Parents in Children’s Pain Experience

Pain experience is influenced by various social, biological, and psychological factors [5]. As outlined in the Social Communication Model of Pain, pain is both an intrapersonal experience, influenced by one’s own dispositions and previous experiences, and an interpersonal experience, occurring within the broader social context [6]. The Affective-Motivational Model of Interpersonal Pain Dynamics highlights the large role parents play in children’s pain experience by integrating emotion and motivation factors that shape the effectiveness of caregiver behaviour (e.g., providing comfort and support to their child in pain) [7]. The observation of pain can elicit a focus on personal well-being (e.g., self-oriented) or towards others’ well-being (e.g., other-oriented), with effective emotion regulation facilitating the latter. Specifically, parents must manage elements of their own emotional response to their child’s pain, including cognitions, emotional states, and physiological responses, in order to accurately interpret and effectively respond to their child’s needs [7]. 

Parents often experience subjective distress, and accompanying physiological activity, when viewing their child in pain, and this emotional reaction can be communicated to their child [6,8,9]. However, rather than solely a response to perceived child pain, physiological activity may also support parent behaviours and responses. Communication between parent and child occurs through various pathways, including facial, vocal, verbal, and gestural; these verbal and nonverbal indices are influenced by internal physiological activity and regulation [10,11]. The most common aspects of adult–child interactions captured during medical procedures include verbal content and gestures, whereas parents’ physiological response has largely been understudied, despite its hypothesized role in supporting both verbal and non-verbal behaviours [10,12,13]. Heart rate (HR) and heart rate variability (HRV; variation in time between consecutive heartbeats) are commonly examined as physiological correlates of emotional experience and regulation [14]. Parents must effectively manage their own distress to better respond to their child’s needs; thus, having an objective index of emotional experience through HR and HRV can provide information about how parents are emotionally responding throughout their child’s pain experience [12,15,16]. 

### 1.2. Heart Rate and Heart Rate Variability

Heart rate, measured in beats per minute, provides a general index of physiological arousal and changes in HR from resting are indicative of emotional reactivity [17]. Caregivers’ HR increases when witnessing their child’s induction of anesthesia [8] and intravenous cannulation [9]. These increases in HR have been associated with higher ratings of children’s preoperative anxiety during anesthesia induction [8] and distress following a venipuncture [9]. 

Heart rate variability at the high or respiratory frequency (also known as respiratory sinus arrhythmia) is posited to reflect social [13] and emotional responding [14,18,19]. HRV can be examined at two levels of analysis: resting (which has been studied more frequently) or phasic (i.e., examining changes in HRV from baseline levels to an event and/or post-event); resting HRV is thought to affect phasic HRV [20]. High resting HRV is generally associated with greater subjective well-being (e.g., better mood, satisfaction with life) [21,22], whereas low resting HRV is associated with poor social and emotional functioning [23,24]. In contrast, understanding increases or decreases in phasic HRV requires consideration of the context [20]. Decreases in HRV tend to occur in response to physical or cognitive stress that does not require executive function, whereas increases in HRV are adaptive in a low-threat context requiring self-regulation [13]. 

To date, one investigation has incorporated parent HRV in the context of pediatric pain [25]. In a laboratory-based study, parents viewed pictures of other people’s children (who had previously completed the pain task their own child was about to undergo) exhibiting varying levels of pain facial expressions, ranging from no pain or neutral to high pain expression. HRV was recorded before and after this viewing task. Findings revealed that HRV decreased from pre-viewing to post-viewing. Notably, HRV was only examined in relation to the solo viewing task; thus, it remains unclear whether a similar pattern of findings would emerge if parents interacted with their own child.

### 1.3. Current Study

The goal of the present study was to add to the understanding of parent internal experience in the context of their child’s pain using a cross-sectional design in a laboratory setting. Parents’ HR and HRV were monitored during three different two-minute time blocks: (1) during a neutral film clip (resting); (2) immediately prior to the child’s cold pressor task (pre-CPT); and (3) immediately following the child’s completion of the cold pressor task (post-CPT). The main objectives were (1) to examine how parent HR and HRV change from resting, to pre-CPT, and post-CPT; and (2) to investigate two factors that could influence HR and HRV across the three phases of the study, including (1) initial HRV and (2) anticipation of difficulty for the child based on their typical response to needle pain. Regarding objective 1, it was hypothesized that parent HR would increase, and HRV decrease, from resting to pre-CPT. This hypothesis was based on research evidencing that observing child pain is often distressing, and previous findings that HR increased before and during child pain [8]. It was also hypothesized that both HR and HRV would return to resting levels following the CPT. Regarding objective 2, given that higher resting HRV is associated with a greater capacity for HRV reactivity [22], it was predicted that parents with higher resting HRV would experience the greatest change from resting to pre-CPT, although these parents would also experience a return to resting post-CPT. Lastly, we predicted that parents who report their child responds negatively to procedural pain would experience the procedure as more stressful, which may be indicated by at least one of the following: higher HR and lower HRV at resting, or a greater increase in HR and decrease in HRV from resting to pre-CPT.

## 2. Materials and Methods

### 2.1. Participants

Participants included 25 children (12 girls and 13 boys) between 7 and 12 years of age (mean (*M*) = 9.44, standard deviation (*SD*) = 1.69) and one of their parents, recruited from the community. The parent sample consisted mainly of White/European (92.0%) mothers (*N* = 24), with a mean age of 42.56 years (range = 32 to 48 years, *SD* = 4.02). In order to participate, the child had to be between 7 and 12 years of age and both child and parent had to have sufficient English proficiency to complete study tasks. One participant who had consumed nicotine two hours prior to the visit and was taking cardioactive medication was excluded from all analyses due to extreme HR and HRV values (*z_skewness_* = 1.49 to 3.66). This pilot study is part of a larger project examining verbal and non-verbal aspects of parent–child interactions. Only data related to parent physiological activity and children’s typical response to painful procedures are reported.

### 2.2. Apparatus

#### 2.2.1. Cold Pressor Task

The Cold Pressor Task (CPT) is a laboratory pain task used to induce mild to moderate levels of pain [26]. Children were first instructed to immerse their hand in a warm water tank (36 °C ± 1 °C) for two minutes to create a standardized baseline of skin temperature. Subsequently, children were asked to place their hand and lower arm in cold water (10 °C ± 1 °C) and to leave it in for as long as they could or until the maximum submersion time of four minutes was reached; participants were unaware of the maximum length of submersion in the cold-water bath (i.e., an uninformed ceiling was used). Water temperature was maintained using a Techne^©^ thermoregulator (Techne Inc., Burlington, VT, USA). This method has been deemed ethically acceptable by researchers, parents, and children and has a rate of adverse events of less than 0.07% [27].

#### 2.2.2. Electrocardiogram

An electrocardiogram (ECG) obtained parent physiological data using a BIOPAC^TM^ MP150 unit and a wireless BioNomadix ECG amplifier (Biopac Systems Canada Inc., Montreal, QC, Canada) acquiring data at 1000 samples per second. Electrodes were placed in a standard Lead II configuration (i.e., an electrode below each collar bone and one ground below the left rib, in an inverted triangle configuration). AcqKnowledge 4.2 software (Biopac Systems Canada Inc., Montreal, QC, Canada) was programmed to identify interbeat intervals (i.e., time between consecutive heart beats) within the ECG recording. Data were then imported into Kubios HRV specialized analysis software (Standard; version 3.0; Kubios Ltd., Kuopio, Finland). HRV guidelines [28,29] and the Kubios HRV User’s Guide [30,31] were followed to quantify HRV. Parental cardiac activity was monitored during three two-minute phases of the study, including: resting, pre-CPT, and post-CPT.

External factors that can influence HRV, such as caffeine and nicotine intake, and cardioactive medication use (e.g., antidepressants, antipsychotics, benzodiazepines, anti-hypertensives) [32] were examined. Bivariate correlations revealed that these factors were unrelated to HR and HRV in the current sample.

#### 2.2.3. Video

Consistent with previous research, a neutral film clip was used to acquire resting HR and HRV data [33]. A two-minute National Geographic Time-Lapse video from YouTube was selected [34].

### 2.3. Procedure

**Figure 1:**
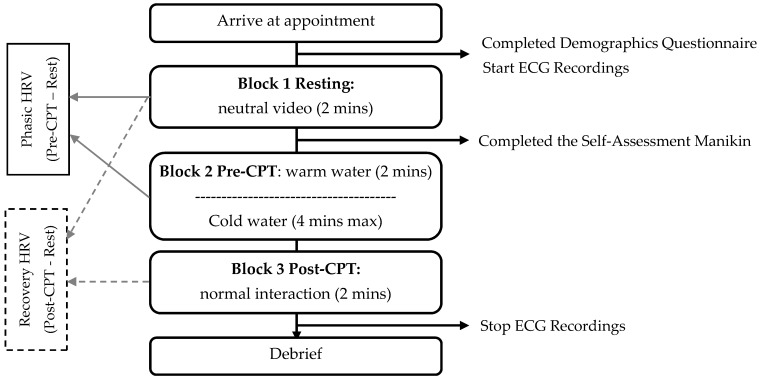
Timeline of procedure. HRV: heart rate variability; CPT: cold pressor task; ECG: electrocardiogram.

Approval from the Research Ethics Board at the University of Guelph was obtained prior to commencing the study (No. 16-12-286). A schematic overview of the study procedure is illustrated in Figure 1. Upon arrival to the lab, parent and child were provided with a brief overview of the study after which parent assent and child assent were obtained before they participated in the study. Parents were fitted with the electrocardiogram equipment and completed the demographic form. As per von Baeyer et al.’s recommendations [26], children were given a juice box to drink prior to completing the CPT to reduce the risk of fainting. Next, child and parent sat and watched a two-minute National Geographic Time-Lapse during the first block of the study (resting block). Parents subsequently rated their arousal and emotional valence in response to the clips and children washed their hands for the upcoming water baths.

The second block of the study consisted of preparation for and completion of the CPT. Parent and child sat facing each other, at a distance between one to three feet. Participants were informed that a knock on the window signalled the child to immerse his/her hand into the warm water tank for two minutes. Parent cardiac activity was monitored during this two-minute interval (pre-CPT block). A second knock on the window informed the child to immerse his/her hand into the cold-water bath. Parent and child were told to interact as they normally would. Once the child voluntarily removed his/her arm from the water or the four-minute maximum time limit was reached, parent and child completed questions regarding their experience as part of the larger project, and the final phase of the study followed. Parental physiological activity was monitored for an additional two minutes following the CPT, while parent and child interacted normally and without instruction (post-CPT block). During each time block, parents were asked to remain seated and minimize their movement in order to reduce movement artefacts.

### 2.4. Measures

#### 2.4.1. Demographics

Data regarding age, sex, language, ethnic and cultural identity were collected through a demographic information form. Parents also rated their child’s usual response to painful needle procedures using a numerical rating scale (0 = negative reaction; 10 = positive reaction). Questions were also included to examine parental caffeine and nicotine intake, and cardioactive medication use, as recommended by Quintana and Heathers [32] when examining cardiac activity; no explicit instructions were given to the parents regarding caffeine or nicotine intake. 

#### 2.4.2. Self-Assessment Manikin

The Self-Assessment Manikin (SAM) [35] was used to measure valence and arousal in response to the video presented during the resting block. The scales range from 1 (happy, stimulated) to 9 (unhappy, relaxed), and a score is provided for both dimensions. 

### 2.5. Data Preparation

Interbeat intervals were first detrended with a smoothness-prior method (i.e., high-pass filter) to remove the very low frequency component (<0.04 Hz). Recordings were visually inspected for artefacts and ectopic heartbeats; no participants breached the convention of 5% threshold for ectopic beats relative to total beats [36]. A threshold-based artefact correction algorithm using cubic spline interpolation was used to correct visually identified artefacts [31]. One waveform required more than 10% of segment (i.e., post-CPT block) editing, and was excluded from analyses [37]. HR and HRV parameters were calculated for the resting, pre-CPT, and post-CPT blocks. Phasic (pre-CPT minus resting) and recovery (post-CPT minus resting) measures were calculated. HR was quantified as beats per minute and HRV was quantified using both time- and frequency-domain methods, as it is recommended to pair HRV frequency analysis with time-domain indices [38]. The time-domain measure of HRV was computed by the root mean square of successive differences (RMSSD) between interbeat intervals, a statistic that is sensitive to high-frequency fluctuations in heart rate [39]. RMSSD values were squared (ms^2^) and natural-log (ln) transformed. The frequency-domain measure of HRV (HF-HRV) was computed by power spectral density analysis which was performed using the Fast-Fourier Transform, with a high frequency (HF) band set at 0.15–0.40 Hz. HF-HRV was natural-log (ln) transformed and reported in squared milliseconds (ms^2^). 

## 3. Results

### 3.1. Pre-Analysis

The current study is a pilot; thus, although findings are reported in terms of traditional statistical significance, it is more meaningful to consider effect sizes and 95% confidence intervals. Effect sizes for correlations are evaluated based on Cohen’s conventions [40]. Means, standard deviations, and the range of HR and HRV measures are presented in Table 1. Descriptive statistics for the change scores of HR and HRV are reported in Table 2. Similar to past research [41], participants rated the film clip neutral to slightly positive (*M* = 3.38, *SD* = 1.25), and relaxing (*M* = 7.00, *SD* = 1.66), ratings appropriate for a “resting” or “baseline” time period. Resting, pre-CPT, and post-CPT measures of HRV demonstrated strong associations (*r* = 0.54 to 0.86, *p* = 0.000 to 0.007). Further, HF-HRV and RMSSD at each time point were strongly correlated (*r* = 0.86 to 0.89, *p* < 0.001; see supplementary material for Tables S1 and S2 for correlations between study variables).

### 3.2. Exploring Parent Cardiac Response during Resting, Pre-CPT, and Post-CPT

**Figure 2:**
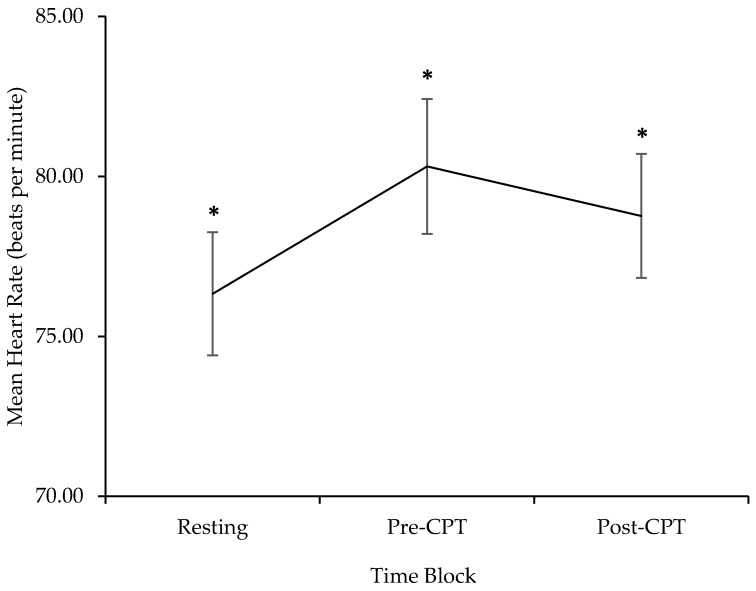
HR activity (beats per minute) during resting, pre-CPT and post-CPT. Bars represent standard error. Asterisks indicate significant differences between blocks, * *p* < 0.05.

A one-way repeated measures ANOVA was conducted to examine differences in parent HR between the three different blocks of the laboratory visit. There was a significant effect of block on HR (*F*(2, 46) = 11.89, *p* < 0.001, partial *η^2^* = 0.34, 95% confidence of interval (CI) [0.17, 0.68]; see Figure 2). Post hoc tests using Fisher’s Least Significant Differences (LSD) revealed an increase in HR from resting to pre-CPT (*M_difference_* = 4.01, standard error (*SE*) = 0.93, *p* < 0.001, 95% CI [2.08, 5.94]). HR during post-CPT decreased from HR during pre-CPT (*M_difference_* = −2.05, *SE* = 0.88, *p* = 0.03, 95% CI [−3.86, −0.24]); however, it remained higher than HR during resting (*M_difference_* = 1.96, *SE* = 0.01, *p* = 0.005, 95% CI [0.67, 3.24]).

**Figure 3:**
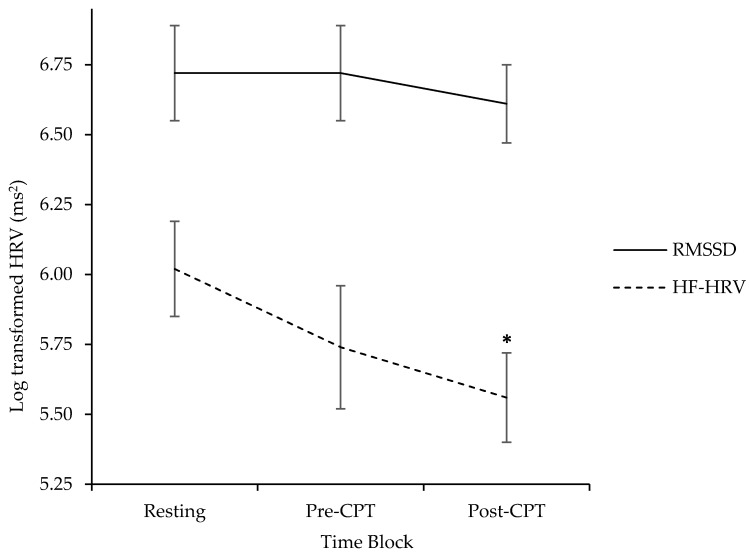
HF-HRV and RMSSD activity (log transformed ms^2^) during resting, pre-CPT and post-CPT. Bars represent standard error. Asterisks indicate significant differences between blocks, * *p* < 0.05.

A second one-way repeated measures ANOVA was conducted to examine differences in parent HF-HRV between the three blocks of the laboratory visit. There was a significant effect of time on HF-HRV (*F*(2, 46) = 3.03, *p* = 0.05, partial *η*^2^ = 0.13, 95% CI [0.00, 0.44]; see Figure 3). Post hoc tests using Fisher’s LSD revealed non-significant differences between HF-HRV pre-CPT and during resting (*M_difference_* = −0.29, *SE* = 0.18, *p* = 0.11, 95% CI [−0.66, 0.08]), and between HF-HRV from pre-CPT to post-CPT (*M_difference_* = 0.12, *SE* = 0.15, *p* = 0.46, 95% CI [−0.20, 0.43]). HF-HRV during post-CPT was lower than HF-HRV during resting (*M_difference_* = −0.41, *SE* = 0.16, *p* = 0.02, 95% CI [−0.74, −0.08]). 

A one-way repeated measures ANOVA was conducted to examine differences in parent RMSSD during the three different blocks of the laboratory visit. There was no significant effect of time on RMSSD (*F*(2, 46) = 0.12, *p* = 0.89, partial *η*^2^ = 0.01, 95% CI [0.00, 0.12]; see Figure 3).

### 3.3. Factors Posited to Affect HR and HRV across the Three Phases of the Study

#### 3.3.1. Resting HRV and Its Association with Phasic and Recovery HRV

Bivariate correlations were conducted to explore resting HF-HRV in relation to phasic HF-HRV and recovery HF-HRV. Resting HF-HRV was not associated with phasic HF-HRV (*r* = −0.14, *p* = 0.50, 95% CI [−0.51, 0.27]), whereas it was related to recovery HF-HRV (*r* = −0.50, *p* = 0.01, 95% CI [−0.75, −0.12]). Similarly, resting RMSSD was not associated with phasic RMSSD (*r* = −0.30, *p* = 0.14, 95% CI [−0.63, 0.11]), although it was associated with RMSSD recovery (*r* = −0.63, *p* = 0.001, 95% CI [−0.82, −0.30]). These results illustrate that parents with higher HRV at rest, experienced the greatest decreases in HRV after the CPT (see Table S2 for the full correlation matrix). 

#### 3.3.2. Children’s Typical Response to Needle Pain

Bivariate correlations were conducted between children’s typical response to needle pain and HR and HRV during resting and change scores across blocks (phasic, recovery). The correlations indicated parent ratings of children’s typical reaction to painful medical procedures (*M* = 4.54, *SD* = 2.17, range = 0 to 9.5; 0 = negative reaction, 10 = positive reaction) were moderately but not always significantly associated with resting HR (*r* = −0.32, *p* = 0.12, 95% CI [−0.62, 0.09]), HF-HRV (*r* = 0.41, *p* = 0.04, 95% CI [09, 0.63]), and RMSSD (*r* = 0.38, *p* = 0.06, 95% CI [−0.0,1, 0.67]). Children’s typical reaction to needle pain was unrelated to phasic HR (*r* = −0.12, *p* = 0.56, 95% CI [−0.49, 0.29]), HF-HRV (*r* = 0.10, *p* = 0.65, 95% CI [−0.31, 0.47]) and RMSSD (*r* = 0.15, *p* = 0.46, 95% CI [−0.26, 0.51]). Typical response to pain was also unrelated to recovery HR (*r* = 0.09, *p* = 0.66, 95% CI [−0.32, 0.48]), HF-HRV (*r* = 0.03, *p* = 0.88, 95% CI [−0.38, 0.43]) and RMSSD (*r* = −0.00, *p* = 0.99, 95% CI [−0.41, 0.40]).

## 4. Discussion

The current pilot study is the first to offer a preliminary examination of parents’ HR and HRV activity as elements of their physiological response in the context of their child’s acute pain. The first objective of this investigation sought to clarify how HR and HRV change over the three time blocks. As expected and consistent with previous findings [8], parents demonstrated increases in HR from resting to before their child’s CPT, suggesting increases in arousal to the anticipation of their child’s pain. Parents’ HR did not return to resting levels during post-CPT and remained significantly higher compared to HR at rest. Of note, although parents did experience significant increases in HR before child pain, their mean values were lower (e.g., 70 to 80 bpm) than what has been typically observed in clinical settings (e.g., 90 to 100 bpm) [8,9]. Thus, parents may not have perceived the CPT to be as threatening or distressing as a venipuncture or anesthesia induction. 

It was also predicted that parent HRV would decrease from resting to pre-CPT, and return to baseline during the post-CPT block. HF-HRV demonstrated a general trend of decreasing from resting, to pre-CPT, and post-CPT; this suggests that parents experienced an emotional response when anticipating and following their child’s CPT. The sizes of these effects in the population may range from small to medium. Decreases in HRV are commonly interpreted as an autonomic response to stress; thus, decreases in parent HRV while anticipating child pain seem logical. However, parent HR remained elevated and HRV lower post-CPT compared to resting, rather than returning to baseline levels as anticipated. Thus, either parents continued to experience stress during the post-CPT period, or the somewhat elevated HR and decreased HRV during this final period may have reflected another process, such as increased interactive engagement with their child post-CPT (e.g., increased attention or executive function demands in the service of communicating) [42]. Of note, decreases in HRV do not necessarily indicate that parents experienced negative emotions; indeed, previous research has demonstrated decreases in HRV in response to both positive and negative stimuli [43]. Therefore, it is possible that the observed increases in HR and decreases in HRV before and after the CPT may reflect general arousal associated with excitement and the dyad viewing the experience as a challenge rather than a threat. This pattern is consistent with a recent investigation [25] demonstrating similar decreases in both HF-HRV and RMSSD from resting to post-viewing. 

With respect to RMSSD-derived indices of HRV, the pattern was generally similar to that of HF-HRV in that it decreased over time, although associations did not achieve statistical significance in this small sample. The general similarity in pattern is unsurprising given the strong correlations observed between phasic HF-HRV and RMSSD (*r* = 0.79 to 0.89) and research indicating both are equivalent indices of HRV [39]. 

A second objective of this work was to investigate factors that may affect HR and HRV across the three phases. Recent research suggests that resting HRV might influence phasic HRV [20], thus resting HRV was examined in relation to changes in phasic and recovery HRV. This pilot study demonstrated that resting HRV was associated with recovery HRV (i.e., while parents interacted normally with their child), although it was not associated with phasic HRV (i.e., immediately prior to child’s pain). That is, parents with higher HRV during the neutral video experienced the greatest decreases in HRV following children’s pain. The size of this effect in the population may range from small to medium. Given that high resting HRV has shown a robust association with positive emotional experience [14], this finding further supports the notion that the observed decreases in HRV following the CPT reflect interactive engagement between parent and child, as opposed to stress regulation. This finding highlights the relevance of considering resting HRV in examining changes in HRV in the context of pediatric pain. 

Lastly, children’s typical response to procedural pain was examined as another potential variable that may predict parents’ HRV. Consistent with the Social Communication Model of Pain [6], parental perceptions of their child’s typical response to painful procedures were related to parent’s HR and HRV at resting, but not to phasic and recovery HR and HRV. Parents who reported that their child usually responds negatively to painful procedures had higher resting HR; although a moderate effect was observed, the wide confidence intervals for this effect indicate that these data are only sufficient to rule out a strong positive association between these variables. These parents also presented with lower resting HRV compared to parents who reported that their child usually responds positively to such procedures. The size of this association in the population may range from small to large. Although the cross-sectional, correlational design precludes definitive comments on directionality, this suggests that parents of children with negative pain experiences may be at a heightened state of arousal and experiencing anxiety long before their child’s actual procedure. A potential clinical implication of this finding is that health professionals may wish to consider children’s previous experience with needle pain as part of a screening tool to identify which parent–child dyads may benefit from treatment strategies.

Biopsychosocial models of pain outline various biological, psychological, and social determinants contributing to the pain experience [44]. In particular, children’s pain occurs in a broader social context of interactions between child and parent. Intrapersonal factors, including previous pain experiences and internal regulation, of both child and parent, are relevant to consider, although parents’ physiological experience has remained largely unknown [6,44]. Taken together, the findings from this pilot study contribute to existing models of pain by highlighting (1) the relevance of measuring parent physiological experience, and (2) the importance of child historical procedural experiences in informing parent cardiac activity at rest.

This novel study has a number of methodological strengths. This is the first investigation to examine both HR and HRV in parents in the context of an acute pain task. As recommended [38], HRV was examined at three different time points (i.e., baseline, event, post-event) which enabled the examination of HRV during resting, and changes in HRV before child pain (phasic), and after child pain (recovery), and using two parameters of HRV (i.e., HF-HRV, RMSSD). Nevertheless, an aspect to be considered in the interpretation of these findings is the pain context in which parent HRV was recorded. The findings from the current study may not generalize to clinical pain, such as needle-pokes, as parent responses may differ in a clinical context. As noted, parent HR was lower than what has been observed during clinical pain and children are given complete control of the pain stimulus during the CPT. Thus, parent and child may not have perceived the CPT to be as painful or fear-inducing as painful medical procedures; children’s historical responses to painful procedures were captured using a single item focused on needles. A more detailed approach which also assesses experiences with experimental pain would be advisable in future research. 

Questions also remain as to whether a preparatory immersion in the warm water bath is needed [26]. In the current study, HR and HRV before the CPT were reported when children had their arm in the warm water, which may have been a positive experience for parents and children and may not reflect pre-pain interactions that may be observed in the context of clinical pain. Future research should examine parents’ physiological response during the cold-water bath. These data were not included in the current study given the smaller sample of children who kept their hand in the cold water for longer than one minute, which is the minimum time needed to acquire HRV data [29]. Incorporating parent ratings of their experience at multiple time points, and in response to both the warm- and cold-water baths, will also help clarify the current findings. However, collecting parent self-reports throughout the procedure may also lead to reactivity effects and increase the time required to complete the study, which can be problematic in clinical contexts. Future investigations may also explore whether a similar physiological profile is obtained when a preparatory immersion in the warm water bath is not included. 

Challenges also exist with acquiring a true baseline measure of parent HRV since they arrive at the laboratory anticipating child pain. Future research would benefit from including a control (i.e., non-pain task) group in order to clarify the extent to which these findings are specific to the pain context. Nevertheless, the current ratings of valence and arousal following the resting block are consistent with previous published ratings [41] and suggest that they perceived the task to be relatively neutral. Future research should examine how parent physiological activity maps onto their self-reported experience and relates to children’s pain outcomes (e.g., pain intensity, fear). In particular, exploring how parent physiological responding translates into observable behavioural responses (e.g., vocalizations, gestures) will be very important. Future research should also explore the interactions among parent self-reported, observed, physiological responses, and child pain outcomes using more complex statistical techniques (e.g., hierarchical multiple regression, moderation analyses, Actor-Partner Interdependence Model). Obtaining multiple post-event recordings can be used to track the progression of HRV following child pain and determine when caregiver HRV returns to resting levels. Finally, findings based on small sample sizes have larger confidence intervals; thus, the actual population means may differ from the estimates found in the current study. Additional research with larger sample sizes is required to develop a more accurate estimate of the effect sizes corresponding to the associations reported here.

## 5. Conclusions

The existing research on parents’ physiological activity in the pediatric acute pain context is limited, despite the observed connections between cardiac activity and emotional experience and regulation. The current pilot study generated knowledge on parents’ HR and HRV activity throughout their child’s completion of a laboratory pain task, and variables that predict this experience. Findings provide preliminary evidence that a multimodal approach to capture parents’ emotional experience in the context of acute pediatric pain would benefit from the inclusion of parent physiological activity.

## Figures and Tables

**Table 1:** Descriptive Statistics of HR, HF-HRV, and RMSSD during Resting, Pre-CPT, and Post-CPT (*N* = 25).

	Resting			Pre-CPT			Post-CPT ^a^		
	*M*	*SD*	Range	*M*	*SD*	Range	*M*	*SD*	Range
HR	76.33	9.59	61.83–97.05	80.31	10.53	61.90–103.99	78.76	9.52	64.31–96.31
HF-HRV	6.02	0.84	4.55–7.36	5.74	1.11	3.26–7.24	5.57	0.80	3.90–7.26
RMSSD	6.72	0.86	4.94–8.06	6.72	0.86	4.39–8.50	6.61	0.68	5.34–7.79

HR = heart rate (reported in beats per minute); HF-HRV = log transformed high-frequency heart rate variability (reported in ms^2^); RMSSD = log transformed root mean square of successive differences (reported in ms^2^); *M* = mean; *SD* = standard deviation. ^a^ *n* = 24 given one data segment that required more than 10% editing.

**Table 2:** Descriptive Statistics of the HR, HF-HRV, and RMSSD Change Scores (*N* = 25).

	Phasic = Pre-CPT − Resting			Recovery ^a^ = Post-CPT − Resting		
	*M*	*SD*	Range	*M*	*SD*	Range
ΔHR	4.00	4.48	−6.15–14.22	1.96	3.05	−2.83 to 8.07
ΔHF-HRV	−0.28	0.85	−2.52 to 1.02	−0.41	0.78	−1.64 to 1.39
ΔRMSSD	0.00	0.67	−2.00 to 1.00	−0.06	0.70	−1.62 to 1.75

Phasic = change score subtracting resting from pre-CPT; Recovery = change score subtracting resting from post-CPT. ^a^ *n* = 24 given one data segment that required more than 10% editing.

## Supplementary Materials

The following are available online at www.mdpi.com/2227-9067/4/11/100/s1. Table S1. Bivariate Correlations Between Children’s Typical Response to Pain and Parent Heart Rate (HR) (*N* = 25). Table S2. Bivariate Correlations Between Children’s Typical Response to Needle Pain and Parent Heart Rate Variability (HRV) (*N* = 25).
