# Supplementary material for: Parent Cardiac Response in the Context of Their Child’s Completion of the Cold Pressor Task: A Pilot Study
Source: Children (Basel). 2017 Nov 21;4(11):100. doi: 10.3390/children4110100 (PMC5704134; doi:10.3390/children4110100)
Supplement: Supplementary file 1 [file children-04-00100-s001.pdf]

Table S1. Bivariate Correlations Between Children's Typical Response to Pain and Parent Heart Rate (HR) (*N* = 25).

	1	2	3	4	5
1. Typical Response to Needle Pain					
2. HR Rest	-.32 [-.63, .09]				
3. HR Pre-CPT	-.34 [-.65, .06]	.90** [.79, .96]			
4. HR Post-CPT[a]	-.29 [-.62, .13]	.95** [.88, .98]	.91** [.80, .96]		
5. HR Phasic	-.12 [-.49, .29]	-.01 [-.41, .38]	.41* [.02, .69]	.11 [-.30, .50]	
6. HR Recovery[a]	.09 [-.32, .48]	-.15 [-.52, .27]	.05 [-.36, .44]	.17 [-.25, .54]	.42* [.03, .71]

HR Phasic = change score subtracting HR during resting from HR during pre-cold pressor task (CPT); HR Recovery = change score subtracting HR during resting from HR during post-CPT. Values in square brackets indicate the 95% confidence interval for each correlation. [a]$n = 24$. *$p < 0.05$. **$p < 0.01$.

Children **2017**, *4*, 100; doi:10.3390/children4110100 www.mdpi.com/journal/children

Table S2. Bivariate Correlations Between Children's Typical Response to Needle Pain and Parent Heart Rate Variability (*N* = 25).

	1	2	3	4	5	6	7	8	9	10
1. Typical Response to Needle Pain										
2. HF-HRV Rest	.41*<br>[.02, .69]									
3. HF-HRV Pre-CPT	.38<br>[-.01, .68]	.65**<br>[.34, .83]								
4. HF-HRV Post-CPT[a]	.46*<br>[.06, .73]	.54**<br>[.17, .77]	.72**<br>[.45, .87]							
5. HF-HRV Phasic	.10<br>[-.31, .47]	-.14<br>[-.51, .27]	.66**<br>[.36, .84]	.40<br>[-.01, .69]						
6. HF-HRV Recovery[a]	.03<br>[-.38, .43]	-.50*<br>[-.75, -.12]	.09<br>[-.32, .48]	.46*<br>[.07, .73]	.59**<br>[.24, .80]					
7. RMSSD Rest	.38<br>[-.01, .68]	.89**<br>[.77, .95]	.48*<br>[.11, .74]	.44*<br>[.05, .72]	-.25<br>[-.59, .16]	-.48*<br>[-.74, -.09]				
8. RMSSD Pre-CPT	.54**<br>[.19, .77]	.76**<br>[.51, .89]	.86**<br>[.71, .94]	.73**<br>[.46, .87]	.38<br>[-.02, .67]	-.02<br>[-.42, .39]	.70**<br>[.42, .86]			
9. RMSS Post-CPT[a]	.47*<br>[.09, .74]	.61**<br>[.28, .81]	.71**<br>[.43, .86]	.86**<br>[.70, .94]	.31<br>[-.11, .63]	.24<br>[-.18, .59]	.59**<br>[.25, .80]	.86**<br>[.70, .94]		
10. RMSSD Phasic[b]	.15<br>[-.26, .51]	-.15<br>[-.51, .26]	.31<br>[-.11, .63]	.25<br>[-.16, .59]	.75**<br>[.50, .88]	.59**<br>[.25, .80]	-.30<br>[-.63, .11]	.36<br>[-.04, .65]	.28<br>[-.12, .61]	
11. RMSSD Recovery[a]	-.00<br>[-.41, .40]	-.47*<br>[-.73, -.08]	.16<br>[-.26, .53]	.30<br>[-.11, .63]	.64**<br>[.32, .83]	.81**<br>[.60, .91]	-.63**<br>[-.82, -.30]	.03<br>[-.38, .43]	.26<br>[-.16, .60]	.75**<br>[.50, 88]

HF-HRV Phasic = change score subtracting high-frequency heart rate variability (HF-HRV) during resting from HF-HRV during pre-CPT; HR-HRV Recovery = change score subtracting HF-HRV during resting from HF-HRV during post-CPT. RMSSD Phasic = change score subtracting the root mean square of successive differences (RMSSD) during resting from RMSSD during pre-CPT; RMSSD Recovery = change score subtracting RMSSD during resting from RMSSD during post-CPT. Values in square brackets indicate the 95% confidence interval for each correlation. [a]$n = 24$; [b]Spearman's correlation coefficient. *$p < 0.05$. **$p < 0.01$
